# Progressive Magnetic Resonance Image Reconstruction Based on Iterative Solution of a Sparse Linear System

**DOI:** 10.1155/IJBI/2006/49378

**Published:** 2006-02-21

**Authors:** Yasser M. Kadah, Ahmed S. Fahmy, Refaat E. Gabr, Keith Heberlein, Xiaoping P. Hu

**Affiliations:** ^1^ Biomedical Imaging Technology Center, Department of Biomedical Engineering, Emory University/Georgia Institute of Technology, Atlanta, GA 30322, USA; ^2^ Biomedical Engineering Department, Cairo University, Giza 12613, Egypt; ^3^ Electrical and Computer Engineering Department, The Johns Hopkins University, Baltimore, MD 21218, USA

## Abstract

Image reconstruction from nonuniformly sampled spatial frequency domain data is an important problem that arises in computed imaging. Current reconstruction techniques suffer from limitations in their model and implementation. In this paper, we present a new reconstruction method that is based on solving a system of linear equations using an efficient iterative approach. Image pixel intensities are related to the measured frequency domain data through a set of linear equations. Although the system matrix is too dense and large to solve by direct inversion in practice, a simple orthogonal transformation to the rows of this matrix is applied to convert the matrix into a sparse one up to a certain chosen level of energy preservation. The transformed system is subsequently solved using the conjugate gradient method. This method is applied to reconstruct images of a numerical phantom as well as magnetic resonance images from experimental spiral imaging data. The results support the theory and demonstrate that the computational load of this method is similar to that of standard gridding, illustrating its practical utility.


Hindawi Publishing Corporation
International Journal of Biomedical Imaging
Volume 2006, Article ID 49378, Pages 1–9
DOI 10.1155/IJBI/2006/49378

### Progressive Magnetic Resonance Image Reconstruction Based on Iterative Solution of a Sparse Linear System

**Yasser M. Kadah,[1,2] Ahmed S. Fahmy,[2,3] Refaat E. Gabr,[2,3] Keith Heberlein,[1] and Xiaoping P. Hu[1]**

[1] *Biomedical Imaging Technology Center, Department of Biomedical Engineering, Emory University/Georgia Institute of Technology, Atlanta, GA 30322, USA*
[2] *Biomedical Engineering Department, Cairo University, Giza 12613, Egypt*
[3] *Electrical and Computer Engineering Department, The Johns Hopkins University, Baltimore, MD 21218, USA*

Received 21 August 2005; Accepted 8 October 2005

Recommended for Publication by Jie Tian

Image reconstruction from nonuniformly sampled spatial frequency domain data is an important problem that arises in computed imaging. Current reconstruction techniques suffer from limitations in their model and implementation. In this paper, we present a new reconstruction method that is based on solving a system of linear equations using an efficient iterative approach. Image pixel intensities are related to the measured frequency domain data through a set of linear equations. Although the system matrix is too dense and large to solve by direct inversion in practice, a simple orthogonal transformation to the rows of this matrix is applied to convert the matrix into a sparse one up to a certain chosen level of energy preservation. The transformed system is subsequently solved using the conjugate gradient method. This method is applied to reconstruct images of a numerical phantom as well as magnetic resonance images from experimental spiral imaging data. The results support the theory and demonstrate that the computational load of this method is similar to that of standard gridding, illustrating its practical utility.

Copyright © 2006 Yasser M. Kadah et al. This is an open access article distributed under the Creative Commons Attribution License, which permits unrestricted use, distribution, and reproduction in any medium, provided the original work is properly cited.

### 1. INTRODUCTION

In this paper, we consider the problem of image reconstruction in magnetic resonance imaging (MRI) without the loss of generality to other tomographic modalities. In the past decade, there has been a great deal of interest and advances in ultrafast MRI. Echo-planar imaging (EPI) is a technique that is widely used for dynamic studies. However, it is limited in speed by how fast the gradients can be switched [1]. To lessen this limitation, nonrectilinear $k$-space trajectories such as spiral trajectory have been used to allow smoother gradient waveforms. This led to a more efficient acquisition process besides the reduction of inhomogeneity and motion artifacts, but image reconstruction from the nonuniformly sampled $k$-space data remains less than optimal in speed and accuracy. Given that conventional MRI relies on discrete Fourier transformation as its reconstruction tool, most existing methods reconstruct the image by gridding, that is, estimating a uniformly sampled $k$-space from the nonuniformly sampled version.

In his seminal work published in 1985, O'Sullivan [2] showed that the ideal resampling kernel is theoretically an infinite Sinc function and that the convolution with such a kernel followed by sampling on a rectilinear grid results in accurate reconstruction. Furthermore, the optimization of an objective function based on a truncated kernel determined that the prolate-spheroidal function is the optimal kernel, which can be well approximated by the Kaiser-Bessel window. This procedure has been utilized in practice as the classical gridding technique for the past two decades. Several papers addressed various aspects of this method, including the optimal selection of the width and form of the gridding kernel for spiral imaging [3], the practical implementation and applications to clinical studies [4, 5], the compensation of sampling density variations [6], the efficient use of computer arithmetic for faster computation [7], and the optimized selection of sampling density compensation factors [8]. The performance of nonrectilinear sampling in the presence of inhomogeneities was also shown to be more robust [9]. Therefore, it is fair to say that gridding is well established in MRI reconstruction. Nevertheless, several issues remain as open questions in using such a method. These issues include the choice of kernel size and shape for different $k$-space trajectories, the possible consequences of undersampling some areas in the $k$-space, and the reduction of the characteristic artifacts associated with gridding.

The above issues led to the development of methods for estimating the rectilinear points from the nonrectilinear ones as a solution to a linear system of equations. This system of equations has a rather large size that is equal to the total number of points. That is, to obtain $N \times N$ rectilinear $k$-space samples, a linear system of size $N^2$ must be solved. Even though the solution to this system would in fact be the optimal solution to the gridding problem, it is not feasible in practice. As a result, a number of attempts have been directed towards obtaining practical approximate solutions to this system of equations [10–13]. Moreover, other authors attempted to estimate the image from the sampled $k$-space using similar techniques [14–18]. Even though these methods offer some advantages over the conventional gridding method, they still have some limitations. First, there is no direct measure of the solution within their formulation and the methods are inflexible in trading off computation time and reconstruction accuracy. The latter is particularly important given the general consensus that gridding techniques are perceived as slow and inflexible in reconstruction time. Second, the computational requirements of the image domain solution techniques [14–18] are still very high for routine use. Therefore, another solution strategy that offers to overcome these limitations is still desired.

In this work, we describe a novel solution to the problem of reconstruction from nonuniformly sampled spatial frequency data. The problem is formulated as a linear system of equations that maps the acquired $k$-space points into the pixel values that represent the image. Even though the linear system is large in size and dense, a simple transformation and thresholding are utilized to convert the system into a sparse form that requires much smaller computational and storage efforts. The sparse system is then solved using the conjugate gradient iterative technique, which enables further control of accuracy versus the computation time. The theory of the new method is described and the results of applying the technique to reconstruct images of a numerical phantom as well as data from a spiral imaging sequence are presented.

### 2. THEORY

Consider $f(x, y)$ as a continuous-space spatial domain intensity distribution and let the available $k$-space (frequency domain) samples be $F(kx_i, ky_i)$, where $i = 0, 1, \ldots, L-1$, and $L$ is the number of samples. The spatial domain can be modeled as piecewise constant function consisting of the sum of shifted gate-like functions representing the pixels of the image at the desired resolution. That is,

$$f(x,y) = \sum_{n=0}^{N-1}\sum_{m=0}^{M-1} \alpha_{n,m} \cdot \Pi(x - x_n, y - y_m). \quad (1)$$

Consequently, the continuous Fourier transform of $f(x, y)$ can be obtained as

$$F(k_x,k_y) = \iint_{-\infty}^{\infty} \sum_{n=0}^{N-1}\sum_{m=0}^{M-1} \alpha_{n,m} \cdot \Pi(x - x_n, y - y_m) \cdot e^{-j2\pi(k_x x + k_y y)} dx\,dy, \quad (2)$$

which reduces to

$$F(k_x,k_y) = \text{Sinc}(w_x k_x) \cdot \text{Sinc}(w_y k_y) \times \sum_{n=0}^{N-1}\sum_{m=0}^{M-1} \alpha_{n,m} \cdot e^{-j2\pi(k_x x_n + k_y y_m)}. \quad (3)$$

Here $w_x$ and $w_y$ correspond to the half of the pixel width in $x$ and $y$ directions, respectively. Given the available arbitrarily located samples in the $k$-space, the above equation leads to a linear system whose equations are obtained by substituting the set of $L$ values for $k_x$ and $k_y$. Hence, the linear system matrix has dimensions of $L$ rows and $M \times N$ columns. The right-hand side vector of this linear system consists of the values of the measured data indexed by $i$. Given this index and from the known locations of the samples in the $k$-space, the system matrix can be computed for a given arrangement of the pixels. The unknowns in this linear system are the pixel intensity values. Specifically, the matrix equation takes the form in (4) and using normalized uniformly sampled spatial points (i.e., $x_n = n \cdot \Delta x$ and $y_m = m \cdot \Delta y$), (4) takes the form in (5)

In order to simplify the solution, we multiply the rows of the system matrix by the $N\cdot M$-point discrete Fourier transform matrix **H** in the following form:

$$H = \frac{1}{\sqrt{NM}} \times \begin{bmatrix} 1 & 1 & \cdots & 1 \\ 1 & e^{-j2\pi/NM} & \cdots & e^{-j2\pi(NM-1)/NM} \\ \vdots & \vdots & \cdots & \vdots \\ 1 & e^{-j2\pi(NM-1)/NM} & \cdots & e^{-j2\pi(NM-1)^2/NM} \end{bmatrix}_{N\cdot M\times N\cdot M}. \tag{6}$$

Note that the inverse of this matrix is simply its Hermitian. This multiplication compacts most of the energy within each row into only a small number of elements in a similar fashion to the transform coding-based compression techniques. As a result, instead of the original dense format of the system matrix, we obtain an equivalent system with a sparse matrix, allowing us to tap into the rich literature of matrix storage and solution techniques in this area. In order to keep the linear system unchanged while taking advantage of this property, we multiply by **H** and its inverse such that

$$\vec{b} = \mathbf{A}\vec{v} = \mathbf{A}\cdot\mathbf{H}^H\cdot\mathbf{H}\cdot\vec{v} = \left(\mathbf{H}\cdot\mathbf{A}^H\right)^H\cdot\vec{V} = \mathbf{M}\cdot\vec{V}, \tag{7}$$

where $\vec{V}$ is the 1D $M\cdot N$-point discrete Fourier transform of the 1D listing of all pixel values in the 2D image and the matrix **M** has most of the energy in each row compacted into only a few elements. In order to make the matrix **M** really sparse, in each row, the element values are sorted by absolute value and only the first few row elements above a threshold are retained. Once this process is completed, the matrix is stored in a sparse matrix format and the conjugate gradient iteration is applied to solve the linear system. A block diagram of the new method is illustrated in Figure 1.

### 3. METHODS

### 3.1. Energy compacting transformation

The energy compacting transformation is implemented by Fourier-transforming the rows of the linear system matrix **A**. The outcome of this transformation is sorted by magnitude and only the largest few elements are retained such that their energy is above a certain fraction of the total energy in each row. Subsequently, such elements are stored by their row and column positions and values as a part of the new sparse linear system matrix. Given that this process is done in a row-by-row fashion, the storage demands for this method are not very high since the huge system matrix need not be constructed or stored.

### 3.2. Sparse matrix manipulation

The storage of the above-derived sparse system can be performed using several techniques. The row-indexed storage method [19], which requires only twice the size of the nonzero elements for their storage, is used in our implementation. With this representation, matrix-vector multiplication operations are only equal in complexity to the number of nonzero elements and always computationally feasible. Also, Hermitian operations are rather simple and do not pose a substantial computational burden.

### 3.3. Conjugate gradient iterative solution

The method of conjugate gradient is used to solve the sparse system. It optimizes the solution of a linear system by removing the error components in a number of directions that span the space of the solution [20]. The original formulation of this iteration requires the system to be real, square, symmetric, and positive definite for the algorithm to work and

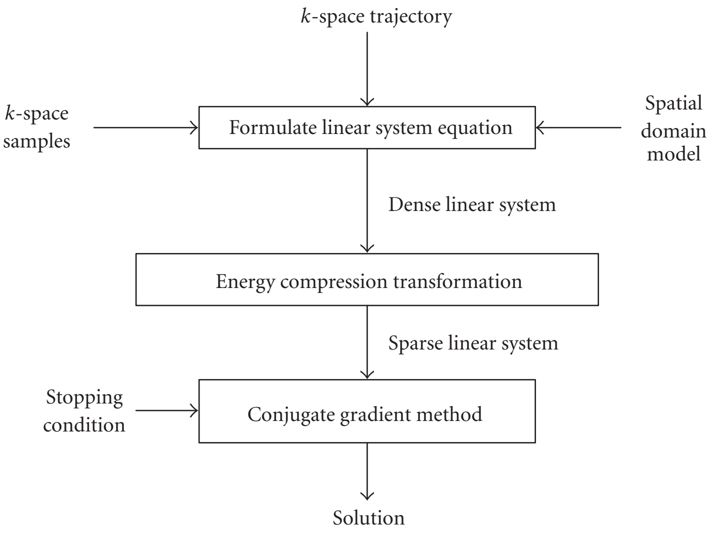

Figure 1: A block diagram of the developed reconstruction method.

(1) Set the initial solution $\vec{x}_0 = \vec{0}$.
(2) Set $\varepsilon$ = required accuracy and/or set the maximum number of iterations.
(3) Compute the initial residual $\vec{r}_0 = A^H \cdot \vec{b}$.
(4) Compute first direction $\vec{p} = \vec{r}_0$.
(5) Compute $\delta = \vec{r}_m^{\,H} \cdot \vec{r}_m / \vec{b}^H \cdot \vec{b}$.
(6) If $\delta < \varepsilon$ or maximum number of iterations reached, then stop.
(7) Let $\vec{q}' = A \cdot \vec{p}$ and $\vec{q} = A^H \cdot \vec{q}'$.
(8) Update solution $\vec{x}_{m+1} = \vec{x}_m + (\vec{r}_m^H \cdot \vec{r}_m / \vec{p}^H \cdot \vec{q}) \cdot \vec{p}$, and update residual $\vec{r}_{m+1} = \vec{r}_m - (\vec{r}_m^H \cdot \vec{r}_m / \vec{p}^H \cdot \vec{q}) \cdot \vec{q}$.
(9) Update direction $\vec{p} = \vec{r}_{m+1} + (\vec{r}_{m+1}^H \cdot \vec{r}_{m+1} / \vec{r}_m^H \cdot \vec{r}_m) \cdot \vec{p}$.
(10) Increment counter $m = m + 1$, and repeat steps (5) through (9) until at least one of the conditions in step (6) is satisfied.

Algorithm 1

provide a unique solution to the system [21]. Here, a variant of this method is applied to solve the normal equations of the system where the system matrix (termed the Grammian matrix) is complex Hermitian, positive definite, and square. The approach based on the delta-criterion [22] has a computational advantage over other methods [9] in the way the stopping condition is checked every step where no further matrix-vector multiplication is involved. In particular, the conjugate gradient algorithm for solving the normal equation $\mathbf{A^H A}\vec{x} = \mathbf{A^H}\vec{b}$ is described in Algorithm 1.

### 3.4. Simulations and experimental data acquisition

The new method was implemented to construct images from numerical phantoms as well as from experimental magnetic resonance imaging data acquired using a spiral imaging sequence. The numerical phantom data were constructed from polar samples of the analytical expression of the Shepp-Logan phantom [14]. The experimental data were acquired on a Siemens Magnetom Trio 3T MRI scanner using a spiral sequence. The spiral sequence had 16 interleaves with 2596 points each to sample a $256 \times 256$ $k$-space matrix using an eight-channel phased array heal coil. The sequence parameters were spin echo, TR/TE = 1000/15 ms, and FOV: 25.6 cm × 25.6 cm. The spiral gradient waveform was designed with a maximum gradient of 40 mT/m and a minimum rise time of 0.5 milliseconds. The spiral gradient readout had a duration of 13 milliseconds per segment and was sampled at 200 kHz.

### 4. RESULTS AND DISCUSSION

Figure 2 illustrates the energy compacting transformation based on simulation. A system matrix was constructed for a $64 \times 64$ gridding problem under polar sampling as shown in Figure 2(a). The resultant system matrix, which has a size of $64^2 \times 64^2$, is shown in Figure 2(b). As can be seen, very few elements are nonzero as opposed to the original dense matrix. The average number of elements per row needed to retain 92% of the kernel energy for this particular case was 3, a significant reduction of elements.

The result of reconstructing the image of the experimental data using conventional gridding is shown in Figure 3(a). This was the best result obtained from this method, using a Kaiser-Bessel window of width 3 and parameter 9. The results obtained using the new method are illustrated in Figures 3(b)–3(d) for energy levels of 90%, 80%, and 70%, respectively. The average numbers of sparse matrix elements/row for these three cases were 3.9, 1.49, and 1.14 elements, respectively. The number of iterations used for all images was 6. As can be observed, the quality of all of them is comparable to that of gridding while having a computational

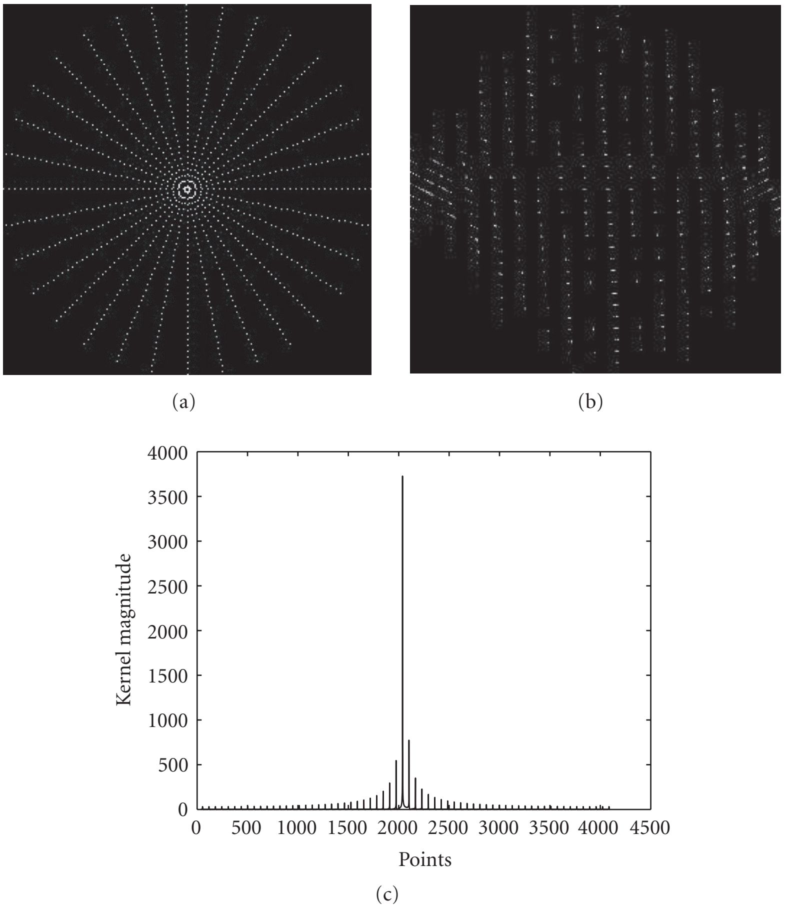

FIGURE 2: Illustration of the energy compacting transformation: (a) example polar trajectory, (b) the constructed system matrix after the energy compacting transformation with only a few nonzero elements, (c) sample row showing only few nonzero peaks.

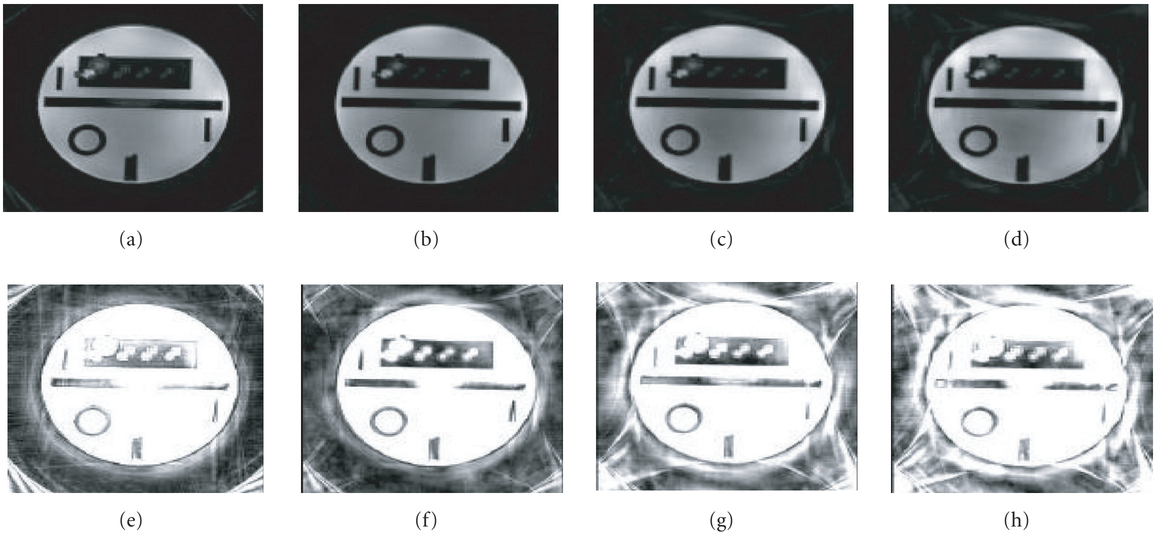

FIGURE 3: Images reconstructed with (a) conventional gridding (best image with optimized parameters), (b-d) new method with energies 90%, 80%, and 70% of the energy retained, respectively. The lower row shows the same images windowed at 10% of the maximum level for each to better illustrate the residual artifacts.

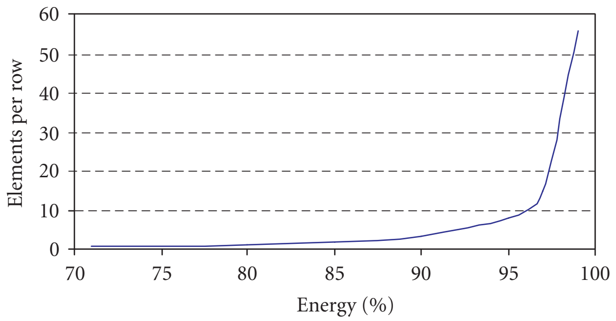

Figure 4: Results from reconstructed images from spiral sampling for image size 128 × 128 showing average number of elements per row for retained energy levels between 70-99%. The number of elements per row increases dramatically after 95% level.

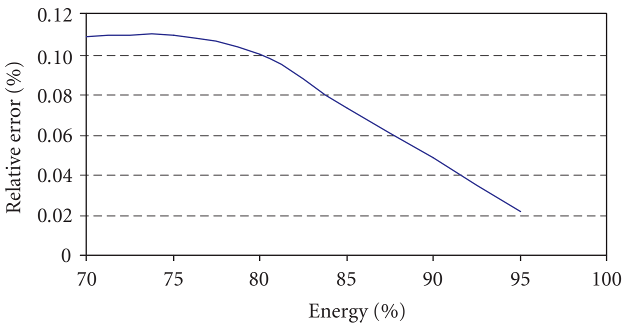

Figure 5: Results from reconstructed images from spiral sampling for image size 128×128 showing reconstruction relative error for retained energy levels between 70- 95%. The number of elements per row increases dramatically after 95% level.

advantage especially for the last image (nearly half the reconstruction complexity of conventional gridding). In Figures 3(e)–3(h), specially-windowed (window center set to 10% of the average intensity) versions of all images are shown to illustrate the residual artifacts. As can be seen, the artifacts differ between conventional gridding and the new method where the latter exhibits vertical and horizontal aliases of the same shape as the object. Note also that the level of residual artifacts increases as the energy retained decreases as expected. The selection of the energy level to be retained controls the average number of elements per row in the sparse matrix conversion process and hence the reconstruction accuracy and complexity. Figures 6 and 4 illustrate the effect of energy retained on the average number of elements per row in the sparse matrix and also the relative error in the reconstructed image. These figures suggest an optimal range between 85% and 95% of the energy to be retained for substantial reduction in complexity while maintaining a sufficiently low error. It is worth noting that the performance of conventional gridding was 8.5 for the equivalent average number of elements per row with an error of 0.37%. Even though the conventional gridding with parameters shown above needs only a single iteration as opposed to the new method, the complexity of multiple iterations of the new method can still be lower than that. Given the significantly higher error, it is evident that the new method has an advantage over the conventional gridding.

To illustrate the rapid convergence of the conjugate gradient iteration, the results of reconstructing the image above with 90% of the energy retained for different numbers of iterations are shown in Figure 5. As can be seen, diagnostic quality reconstruction can be achieved with as low as 2-3 iterations. The convergence of this experiment is shown in Figure 7 where the delta-criterion was computed and used as the error measure. The error converges very fast to reach a level of approximately $10^{-6}$ in the 10th iteration. Note that the oscillatory form of the convergence is mainly due to the fact that the problem solved is an approximation to the original one.

It should be noted that the size and location of the large matrix elements that are selected to achieve the kernel energy percentage varies from one row to another. This means that the kernel used to perform the mapping is spatially varying. We note also that the truncation using this method is optimal in the least-squares sense. The selection using this method avoids having the user choose more specific parameters of reconstruction like window size or neighborhood definition like other techniques [3, 10]. Instead, a single parameter is used as a quality measure (the energy percentage) while another is used as a computation time control (number of conjugate gradient iterations).

The computational complexity of the new method is $O(N^2)$ to obtain the solution to the linear system in addition to one $N^2$-point 1D FFT required afterwards. This computational complexity is well within the range of that

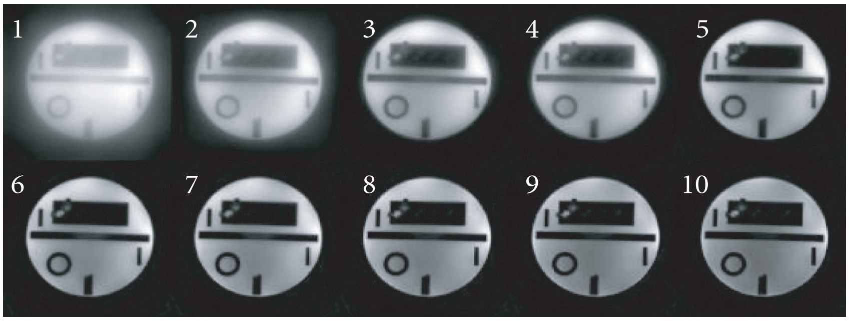

Figure 6: Reconstructed images from spiral sampling of real phantom data for image size 256 × 256 showing output from iterations 1 through 10. We start to see diagnostic quality images after only a few iterations.

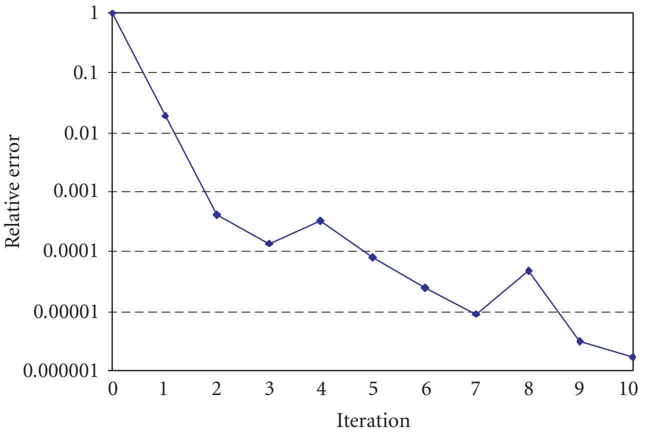

Figure 7: The convergence characteristics of the iteration used in the previous figure. As can be observed, only a few iterations are needed to substantially reduce the reconstruction error.

for conventional gridding methods as well as gridding techniques based on localized SVD solution. This complexity is several orders of magnitude below other iterative reconstruction techniques [14–18] given its linear dependence on the number of points in the image in the matrix inversion part. The ability of the user to control the reconstruction time is a unique feature in this method. This allows a quick, almost real-time computation of images for fast viewing by the radiologist. Furthermore, it allows the radiologist to increase the accuracy of the reconstruction of a selected image at will simply by allowing additional iterations to run. Given the low complexity of iterations, such process can be performed during image viewing using console control just like zooming or gamma curve selection with virtually no noticeable delay.

The method of conjugate gradient has a number of advantages. First only a few iterations are usually required to reach a good accuracy for the solution. Another advantage is that the solution accuracy can be traded off with computation time rather flexibly. This allows the method to be customized for the particular application at hand by selecting a predetermined number of iterations that correspond to the desired computation time. If a high accuracy is desired, an efficient implementation of this method may rely on a measure of the solution update in such a way that the stopping criterion is to have an update that is insignificant compared to the present solution.

The potential applications of the new method are several. The most obvious is its use to generate real-time reconstruction of spiral imaging sequences. This can be useful in applications such as functional MRI (fMRI) where real-time activation detection and display are increasingly more available on commercial scanners. Another application is to generate fast spiral navigator reconstructions for use with segmented acquisition sequences. The new method can potentially be modified to include special reconstruction procedures such as field inhomogeneity correction where a distortion in the spatial domain needs to be taken into account. In this case, the reconstruction and artifact suppression can be achieved in a single step. Applications also extend to other modalities such as computerized tomography (CT). Further investigation of such applications is needed to assess their value in practice.

### 5. CONCLUSIONS

A new method for image reconstruction from nonuniformly sampled spatial frequency domain data is presented. This method provides the means for solving the reconstruction problem iteratively based on an efficient algorithm, which permits the solution of the large linear system of the problem after conversion to sparse form. A unique aspect of this technique is the availability of explicit reconstruction error measures that can be monitored and controlled for customized performance. More work is needed to investigate the use of the new method in practical clinical applications.

### ACKNOWLEDGMENTS

This work was supported in part by the NIH (Grants RO1EB00331 and RO1EB002009), the Georgia Research Alliance, and the Whitaker Foundation.

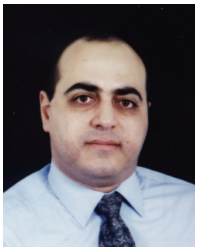

**Yasser M. Kadah** received his B.S. and M.S. degrees from the Biomedical Engineering Department Cairo University in 1989 and 1992, respectively. He received his Ph.D. in biomedical engineering from the University of Minnesota in 1997. He worked as a post-doctoral fellow at the Center of Magnetic Resonance Research at the University of Minnesota in 1998. He became an Assistant Professor of biomedical engineering at Cairo University from 1998 to 2003. He was a Research Associate with the Biomedical Imaging Technology Center at the Biomedical Engineering Department, Emory University and Georgia Institute of Technology, from December 2002 till July 2004. He is currently an Associate Professor of biomedical engineering at Cairo University. In 2002, he was selected to receive the E.K. Zaviosky stipend from the International Society of Magnetic Resonance in Imaging (ISMRM). He was also elected a Senior Member of the Institute of Electrical and Electronic Engineers (IEEE) in 2003. He is currently an active Member of ISMRM and the International Society of Optical Engineering (SPIE). His research interests include medical imaging and in particular MRI and ultrasound, and multidimensional signal processing for biomedical applications.

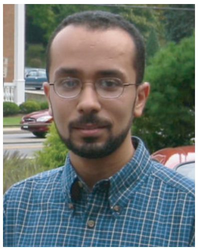

**Ahmed S. Fahmy** obtained a bachelor and a Master degrees in biomedical engineering from Cairo University, Egypt, in 1997 and 2001, respectively. In August 2001, he moved to the United States where he joined the Electrical and Computer Engineering Department in Johns Hopkins University, Maryland, USA, as a Research Assistant. He got a Master degree in electrical engineering from Johns Hopkins University. Mr. Fahmy is currently a Ph.D. candidate in the Electrical and Computer Engineering Department, Johns Hopkins University. His current research interests include multidimensional signal processing, pulse sequence programming, and MR cardiac imaging.

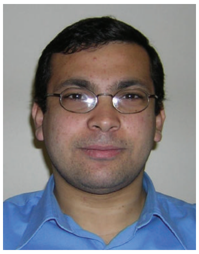

**Refaat E. Gabr** received his B.S. and M.S. degrees in biomedical engineering from Cairo university, Egypt, in 2000 and 2003, respectively. He was appointed as a Teaching Assistant in the same university in 2000, where he participated in teaching random signal analysis, data structures, and analog and digital electronics. He was a part-time engineer in Egypt in the period 2002-2004, designing and implementing control and automation systems for industry. In 2004, he joined the Department of Electrical and Computer Engineering at the Johns Hopkins University as a Ph.D. student, where he is doing research in magnetic resonance imaging. Refaat Gabr research interests include signal and image processing, medical imaging, magnetic resonance spectroscopy, and magnetic resonance imaging.

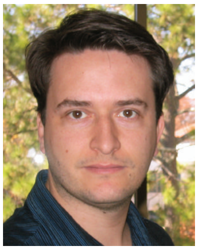

**Keith Heberlein** obtained his Ph.D. in biomedical Engineering from the University of Minnesota in 2003. Following graduation, he joined the Biomedical Imaging Technology Center at Emory University and Georgia Tech as a Research Associate under the supervision of Xiaoping Hu. His research focus is on the engineering of MRI technology for medical and research applications. He has authored or co-authored 8 peer-reviewed journal articles and 19 conference papers. His undergraduate work was completed in 1997 at the University of Florida in the Department of Engineering Science and Mechanics. Dr. Heberlein is a Member of the International Society for Magnetic Resonance in Medicine and has reviewed manuscripts for Magnetic Resonance in Medicine.

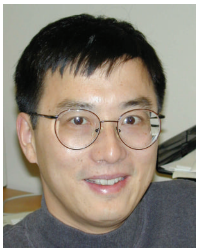

**Xiaoping P. Hu** obtained his Ph.D. in MR physics from the University of Chicago in 1988. After spending 2 years as a Research Associate at the University of Chicago, he joined the faculty of the Department of Radiology at the University of Minnesota, where he was an Assistant Professor (1990-1994), an Associate Professor (1994-1998), and a Full Professor (1998-2001). In January 2002, he moved to Emory University/Georgia Institute of Technology to become a Professor of biomedical engineering, a Georgia Research Alliance Eminent Scholar and the Director of the Biomedical Imaging Technology Center. His research interest lies in the development and biomedical application of magnetic resonance imaging. To date, Dr. Hu has authored or co-authored 118 peer-reviewed journal articles and holds 4 MR patents. Dr. Hu served on the editorial board of Magnetic Resonance in Medicine in 2002-2004; he is currently a Deputy Editor of Magnetic Resonance in Medicine. He is also an Associate Editor of IEEE Transactions on Medical Imaging (1994-present), managing the review of MRI related manuscripts. In 1998 and 2001, Dr. Hu served as a Guest Editor of NMR in biomedicine for its special issues on fMRI. Dr. Hu is a Senior Member of the IEEE. Dr. Hu was named an RSNA Researcher Scholar in 1991 and a Fellow of the International Society for Magnetic Resonance in Medicine in 2004.

### REFERENCES

[1] E. M. Haacke, R. W. Brown, M. R. Thompson, and R. Venkatesan, *Magnetic Resonance Imaging: Physical Principles and Sequence Design*, John Wiley & Sons, New York, NY, USA, 1999.

[2] J. D. O'Sullivan, "A fast sinc function gridding algorithm for Fourier inversion in computer tomography," *IEEE Transactions on Medical Imaging*, vol. 4, no. 4, pp. 200–207, 1985.

[3] J. I. Jackson, C. H. Meyer, D. G. Nishimura, and A. Macovski, "Selection of a convolution function for Fourier inversion using gridding," *IEEE Transactions on Medical Imaging*, vol. 10, no. 3, pp. 473–478, 1991.

[4] C. H. Meyer, B. S. Hu, D. G. Nishimura, and A. Macovski, "Fast spiral coronary artery imaging," *Magnetic Resonance in Medicine*, vol. 28, no. 2, pp. 202–213, 1992.
[5] H. Schomberg and J. Timmer, "The gridding method for image reconstruction by Fourier transformation," *IEEE Transactions on Medical Imaging*, vol. 14, no. 3, pp. 596–607, 1995.
[6] J. G. Pipe and P. Menon, "Sampling density compensation in MRI: rationale and an iterative numerical solution," *Magnetic Resonance in Medicine*, vol. 41, no. 1, pp. 179–186, 1999.
[7] J.-R. Liao, "Real-time image reconstruction for spiral MRI using fixed-point calculation," *IEEE Transactions on Medical Imaging*, vol. 19, no. 7, pp. 690–698, 2000.
[8] V. Rasche, R. Proksa, R. Sinkus, P. Bornert, and H. Eggers, "Resampling of data between arbitrary grids using convolution interpolation," *IEEE Transactions on Medical Imaging*, vol. 18, no. 5, pp. 385–392, 1999.
[9] Y. M. Kadah and X. P. Hu, "Algebraic reconstruction for magnetic resonance imaging under B0 inhomogeneity," *IEEE Transactions on Medical Imaging*, vol. 17, no. 3, pp. 362–370, 1998.
[10] D. Rosenfeld, "An optimal and efficient new gridding algorithm using singular value decomposition," *Magnetic Resonance in Medicine*, vol. 40, no. 1, pp. 14–23, 1998.
[11] H. Moriguchi, M. Wendt, and J. L. Duerk, "Applying the uniform resampling (URS) algorithm to a lissajous trajectory: fast image reconstruction with optimal gridding," *Magnetic Resonance in Medicine*, vol. 44, no. 5, pp. 766–781, 2000.
[12] H. Moriguchi and J. L. Duerk, "Modified block uniform resampling (BURS) algorithm using truncated singular value decomposition: fast accurate gridding with noise and artifact reduction," *Magnetic Resonance in Medicine*, vol. 46, no. 6, pp. 1189–1201, 2001.
[13] H. Sedarat and D. G. Nishimura, "On the optimality of the gridding reconstruction algorithm," *IEEE Transactions on Medical Imaging*, vol. 19, no. 4, pp. 306–317, 2000.
[14] R. Van De Walle, H. H. Barrett, K. J. Myers, et al., "Reconstruction of MR images from data acquired on a general nonregular grid by pseudoinverse calculation," *IEEE Transactions on Medical Imaging*, vol. 19, no. 12, pp. 1160–1167, 2000.
[15] B. P. Sutton, D. C. Noll, and J. A. Fessler, "Fast, iterative image reconstruction for MRI in the presence of field inhomogeneities," *IEEE Transactions on Medical Imaging*, vol. 22, no. 2, pp. 178–188, 2003.
[16] Q. H. Liu and X. Y. Tang, "Iterative algorithm for nonuniform inverse fast Fourier transform (NU-IFFT)," *Electronics Letters*, vol. 34, no. 20, pp. 1913–1914, 1998.
[17] J. A. Fessler and B. P. Sutton, "Nonuniform fast Fourier transforms using min-max interpolation," *IEEE Transactions on Signal Processing*, vol. 51, no. 2, pp. 560–574, 2003.
[18] T. Strohmer, "Computationally attractive reconstruction of bandlimited images from irregular samples," *IEEE Transactions on Image Processing*, vol. 6, no. 4, pp. 540–548, 1997.
[19] W. H. Press, B. P. Flannery, S. A. Teukolsky, and W. T. Vetterling, *Numerical Recipes in C: The Art of Scientific Computing*, Cambridge University Press, Cambridge, UK, 2nd edition, 1992.
[20] G. H. Golub and C. F. Van Loan, *Matrix Computations*, The Johns Hopkins University Press, Baltimore, Md, USA, 2nd edition, 1989.
[21] M. R. Hestenes, *Conjugate Direction Methods in Optimization*, Springer, Heidelberg, Germany, 1980.
[22] K. P. Pruessmann, M. Weiger, P. Bornert, and P. Boesiger, "Advances in sensitivity encoding with arbitrary $k$-space trajectories," *Magnetic Resonance in Medicine*, vol. 46, no. 4, pp. 638–651, 2001.

